# Acknowledgement to reviewers

**DOI:** 10.1530/EC-11-A1

**Published:** 2022-01-31

The editorial board wishes to thank the following individuals who have helped to review manuscripts for *Endocrine Connections* during 2021. Their assistance is gratefully acknowledged.

M F Abalem

S Adam

J S Adams

R Afrisham

S Agosto

S Agosto Salgado

S Ahmadi

B-C Ahn

S Akin

K Alexandraki

S Allehdan

J F M de Almeida

M Almeida

A Al-Mutair

B Altieri

A D Anastasilakis

N H Andersen

R A Anderson

M Andreassen

T Angell

A Angelousi

G Arnaldi

A P Athanasoulia-Kaspar

P Attila

F Azizi

C Badiu

J H Baek

O R Bandapalli

J P Banga

A Baniahmad

N Banks

T Barber

M Barbieri

M Barbot

M Barczynski

A Bartelt

S Basu

C Bendinelli

S Bernardi

N Berruien

J-P Bertocchio

F Beuschlein

E Bhatia

H Biebermann

A Bikas

I Bilic-Curcic

M Bjerre

C S Blesson

M Bluher

A Boelen

F Bogazzi

S J Bonnema

V Boraska Perica

B J Boucher

J E Bowe

P Breining

K Brix

J J Broseta

F Cadario

X Cai

M Calan

G Callo

P G Calo

B Cangiano

R Cannarella

M C Cantone

L Canu

Y Cao

C Carlberg

E Castellano

M R Castro

E Cavalier

J Cazarin

F Ceccato

S Cetinkaya

L F Chan

K Chapman

C Chatzakis

C-J Chen

T Chen

W Chen

X Chen

C-L Cheung

M S Cheung

D Chia

S W Cho

Y-M Choi

A Chopra

A C Chrisoulidou

G Chrousos

H K Chung

K W Chung

S Ciardullo

C Ciccacci

A Cinzia

A Clark

M Clerici

M S Cooper

P Cramon

G Curic

G Cvijovic

J B Cwikla

A Cypess

M da Rocha

P K Dabla

C Dariane

F D’Aurizio

L E Davidson

A De Bray

M De Castro

L C G de Graaff

W W de Herder

F de Zegher

M Debono

S A DeFlorio-Barker

J Deinum

L C Delfino

K Dhatariya

G Di Dalmazi

J W Dietrich

N Ding

S Domenice

X Dong

R M Dores

K Duffin

A Duleba

W C Duncan

K Dziopa

G Effraimidis

G Eisenhofer

Y Elhassan

R Elisei

C Ellervik

A El-Remessy

W P J den E Elzen

S Faghihi-Kashani

B Falkowski

Y Fan

E Ferrante

A Ferreira

S P Fitzgerald

C E E Fluck

F Fogacci

L A Fondjo

P A Foster

A Franco

S Frey

G Fruhbeck

J Frystyk

J Funder

F Gabalec

S Gaberscek

S A Gabr

G Gabriel

J Gajewska

A Gambineri

M Gandhi

S Gao

D Gąsior-Perczak

J Gauer

C Gazzaruso

N Georgopoulos

M Ghamari-Langroudi

D Gianfrilli

S Giannini

O Gimm

M Girndt

M Giroud

A Gjesing

J M Gohlke

G Goliasch

R Gorrigan

P Goundan

W B Grant

C Grasemann

L Gregory

G E Grieco

S Groß

A Grossman

U Gruntmanis

H Guan

G G Guaragna-Filho

F Guerrero-Romero

J Gunter

P Guo

X Guo

F Hadziselimovic

T Hamada

B Han

D J Handelsman

Z Hannoush

L B Hansen

R J Hart

Z Hassan-Smith

F H Hawkins Carranza

J He

X He

M Hecking

J Hertel

T Hökfelt

R Holaj

M Holick

P Holmager

S-H Hong

S Howard

S-M Hsia

C Hu

Y M Hu

P Huang

R Huang

C-C Huang

Y Huang

M S Huhtala

G Hundemer

H Inaba

W J Inder

S Ing

S Ippolito

N Ito

Y Ito

S Jain

J A M J L Janssen

B Jarzab

M J Jeon

P Jerry

P Jezek

C Jiang

N Jiang

Y S Jo

W Johnson

T Johnson

S Jonard

A P Jorgensen

C C Juhlin

D Kajdaniuk

K Kakudo

U Kampmann Opstrup

N Kapoor

C Karadeniz

O Karapanou

M Karownik-Lewinska

D Kastelan

H Kazaure

E X Keller

T Kessler

M Khan

C M Khoo

F W Kiefer

B H Kim

B-J Kim

C Kim

K Kim

M Kim

M H Kim

R Kim

S Kim

S H Kim

S Y Kim

T Y Kim

W G Kim

K S Kirkegaard

A Klisic

G Klöppel

S Knorr

T Kocjan

J Koehrle

N Koibuchi

S Kondeti

I Konrade

T Korevaar

F Koromani

B Kos-Kudła

J Krajewska

M S Kuchay

R Kumar

I Kurihara

A Kuś

H Kwon

A Kyriacou

S La Vignera

G M Lambert-Messerlian

M Lantz

C R Lanzi

A Larsson

Y S Lee

J Lei

P Lei

C Letizia

M Levine

M Levy

F Levy-Khademi

B Li

G Li

J Li

X-I Li

Y Li

D J Lim

J-M Lin

W Lin

J Liu

J L Liu

L Liu

S Livadas

P Locantore

L Locati

L Lösser

Y Louwers

G Lu

X Lu

J Ma

A Machens

D Macut

B Madeo

A Madsen

P Makras

M Mannelli

J L Mansur

G Mantovani

E Mantzou

M Marcelino

C Marcocci

C P Marginean

M Marino

I L Martinez de LaPiscina

L Martinez-Mota

J Martinez-Quintana

I J Martins

G Mastorakos

H Masutani

N Matikainen

A Matrone

R Mckenzie

B Meczekalski

L Mehran

O Meijer

B B Mendonca

M Mercado

B Merino

D Merlotti

I Messinis

K Michalakis

R Mihai

B S Miller

G Mintziori

P Miotto

A Mittal

M Mogl

V Mohan

J H Moon

S Mukherjee

S Mukhopadhyay

A Mukuve

I Muller

P G Murray

C Muschitz

E Muzurović

A Nagai

M Nagao

D B Naik

S Nakaji

M Nazari

R Negro

J P Nicola

E J Nieveen van Dijkum

O Nilsson

M Nixon

S Nochaiwong

G Ntali

H F Nyström

M O`Connell

S Oberfield

M Oczko-Wojciechowska

S Olesen

M Opalinska

J Oriola

M J Ornstrup

V Ostadmohammadi

K Ottarsdottir

O O Oyewole

D Pach

R Pal

A Palermo

G Paltoglou

D Papadimitriou

M Papaleontiou

I Papassotiriou

T Pappa

Y J Park

R Paschke

V Patil

E B Pedersen

N S Pellegata

J Pepe

C Perego

J Perkins

F Persson

V Petrenko

J Pettifor

V Peyret

M Peyrou

A Piekielko-Witkowska

L Pirola

P Pludowski

M Podolsky

R M Pop

M Portero Otin

A P Prats-Puig

K Prentice

S Puglisi

L L Pyle

H Qu

R Quinton

G Radetti

X Ran

A E Ratajczak

M Rauner

P Ravn

M Read

E Rebelos

M Reed

V Reeve

J Rege

L Rejnmark

U Renner

G Reusz

R A Rey

S Riaz

V B Ribeiro

E Rios

V Rochira

S Rodgers

A Rodriguez

A Rojas Rubio

J A Romijn

G Romina

D Romualdi

B Roozendaal

R J Ross

M Ruchala

J Rüdebusch

R Ruggeri

H Russ

J D Safer

Y Saisho

K Saltiki

A Sanchez-Garcia

P E Sanchez-Hernandez

V Sarapura

M Sargin

R M Sargis

F C C Sasso

Y Sato

C Satterfield

D Saxon

M Scatolini

C Schlein

R Schönbauer

J Schopohl

F Schulze

P Schwarz

Y Schwarz

R Scragg

J Segovia

D Sekar

A Sen

M Senoymak

A Sertedaki

V N Shah

S Shalet

Z Shan

X Shen

Y Shi

A Shinkov

A Shpakov

K Siddiqui

S Sidhu

Z Siklar

L F G Silveira

O Silverman

W F Simonds

M Simopoulou

G Singh

R Singla

A Skalniak

T J Smith

P Soares

M Sodersten

Y Sohma

S Y Sohn

L A Solbiati

D E Song

G Speer

K Stamatelopoulos

T L Stanley

M Stasiak

J Stegbauer

R Sterenborg

K Stochholm

C J Strasburger

J Su

N Sukor

S A Sulaiman

L Sun

S Suzuki

E Szczepanek-Parulska

S Szlapinski

D Taieb

J Tamblyn

M Tanaka

Y-X Tao

W Teng

X Teng

E Thiis-Evensen

J W Tomlinson

K Tommerdahl

F Tonelli

B Torlinska

C Toumpanakis

P Touraine

F Trepiccione

M Trofimiuk-Mouldner

S Trompet

M Trovato

V Tsang

A N Tsartsalis

O Tsave

D Tsilingiris

J Tuckermann

P Tzoulis

S Uchino

G A Ueland

U Ünlütürk

B Vaidya

F Vaisman

N Valdes

A Valent

A J Van der Lely

E Vasilopoulou

D A Vassiliadi

L Vergnes

E T Vestergaard

G Vila

A Vinagre

E Visser

G Vitale

M H Viuff

M R Vriens

C A Wagner

H Wang

P Wang

Q Wang

X Wang

XS Wang

Y Wang

Z Wang

T Watt

D J Waxman

S M Webb

D Weber

J Wen

Y Weng

U Wenzel

S J Whiting

W M Wiersinga

L E A Wildemberg

H S Willenberg

C Williams

J Williams

T-A Williams

E M Winter

M Wojcik

B Wu

J-N Wu

H Xiao

S Xu

T Xu

S Yadav

X X Yan

C Yang

G Yang

J Yang

W Yang

X Yang

M Yavropoulou

C Yayla

S Ye

J Yin

H Yoon

J S Yoon

S Yoshino

L Zabuliene

T T Zampieri

D E Zantut-Wittmann

M Zatelli

H Zhan

H Zhang

Y Zhang

H Zhao

J Zhao

J-j Zhao

W Zhao

Y M Zhao

Y Zheng

J Zhou

K Zhou

H Zhu

L Zhu

S Zórad

D Zwanziger

